# Pandemic effects to autonomous vehicles test operations in California

**DOI:** 10.1371/journal.pone.0264484

**Published:** 2022-03-10

**Authors:** Adrian Chen Yang Tan

**Affiliations:** Project and Supply Chain Management, Penn State University New Kensington, New Kensington, Pennsylvania, United States of America; Southeast University, CHINA

## Abstract

Companies developing automated driving system (ADS) technologies have spent heavily in recent years to conduct live testing of autonomous vehicles operating in real world environments to ensure their reliable and safe operations. However, the unexpected onset and ongoing resurgent effects of the Covid-19 pandemic starting in March 2020 has serve to halt, change, or delay the achievement of these new product development test objectives. This study draws on data obtained from the California automated vehicle test program to determine the extent that testing trends, test resumptions, and test environments have been affected by the pandemic. The importance of government policies to support and enable autonomous vehicles development during pandemic conditions is highlighted.

## Introduction

A successful development of automated driving system (ADS) technology will achieve a major milestone to expand the capabilities of future supply chain logistics. Automation in road vehicles can significantly improve productivity, agility, and safety in logistics [1]. Primarily, this is because automation will reduce or eliminate the direct use of the human element in vehicular operations. The highest recurring cost in logistics is that of human labor, and human resources cost are projected to only continue to increase in the future [2]. It has been possible to introduce various beneficial automation innovations to improve the productivity in different areas in logistics such as materials handling, warehousing, inventory management, or order picking. However, implementing automation to vehicular operations is a vastly more complex and difficult undertaking because of the need for these vehicles to operate alongside other road-users on public roads. There exist legitimate concerns on whether computers, software and sensors can reliably operate automated vehicles [3–6], and whether they may safely share the use of road space with other vehicles, cyclists, or pedestrians [7,8]. In turn, companies developing automated vehicles technologies have sought to allay skepticism over the utility of ADS technology by conducting extensive software simulation, modelling, as well as live-testing of vehicle prototypes on roads [9–11].

Typically, field testing of prototypes is a critical stage in the new technology development process. Field tests are particularly effective at detecting unexpected divergences from intended to actual usages, and identifying interventions that can correct technological development paths [12]. Through field tests, companies can seek to meet development objectives to confirm functionality to specifications, assess risks, determine customers’ acceptance levels, perform subsystems integration, and guide error debugging [13,14]. Extensive and well executed test programs can serve to allay public fears, and instill confidence in new technologies in stakeholders.

SAE International defines an ADS as the collective hardware and software capable of performing the entire dynamic driving task of a vehicle on an ongoing basis. SAE International defines six levels of automation for vehicles that are also adopted by the US Department of Transportation. These levels of automation range from a Level “0” which means no driving automation and where a human driver needs to handle all driving tasks regardless of the inclusion of warning or intervention systems, all the way up to Level “5” which means complete driving automation without restrictions [1,15,16]. Companies in the United States are currently testing automated vehicles on public roads of at least Level “4” automation, which is defined as the automation level in which an automated vehicle has to be capable of sustained driving performance as specified without any expectation that a human being will need to respond to a request to intervene in the driving function [15,17,18].

The State of California, via a series of regulations passed in 2014, and updated in 2018, allows companies to test automated vehicles that are still under development on public roads. During such tests, these vehicles move in autonomous mode through live traffic without human input, though they are monitored by test drivers who can take over the driving function if an automated vehicle encounters an unexpected issue it cannot handle, or if a test driver makes a judgement call to stop the test drive due to a developing and problematic road situation [19]. A company that registers with the Department of Motor Vehicles, or DMV, in California to test automated vehicles is subject to the regulations published in the California Code of Regulations under Title 13, Division 1, Chapter 1, Article 3.7 “Testing of Autonomous Vehicles” [20,21]. These companies are required to provide annual test disengagements and mileage information, and reports of collisions as they occur to the DMV for public disclosure. Since September 2014, 51 companies have been entered into the DMV test register, though many subsequently exit the test program. As of end-2020, 36 test companies are currently registered in California to perform tests on automated vehicles with drivers present [19]. Since 2018, the State of California also permit another category of automated vehicle testing without drivers present. That category is not covered in this paper due to the very limited data currently available [22].

The need for thorough field testing is particularly necessary for development such as ADS technology, which cutting-edge novelty inherently involves higher risks of failures [14]. The field test process for ADS technology varies from company to company. However, the basic procedure is likely to be similar to these abbreviated points below provided by the test company GM Cruise operating in California [23].

- Gather data by driving fleet of autonomous vehicles (AV) throughout San Francisco.
- Analyze the data for new events, gaps, and opportunities to improve.
- Update the AV code and simulation code.
- Test the new AV code in simulation.
- Deploy code to AV fleet, and then go back to point one.

The sequence of these guidelines points highlights the importance of field tests as being integral to the process of ADS development, and thereby cannot be completely replaced by simulation testing or in-house track testing alone.

The sudden emergence of the Covid-19 virus in 2019, and its subsequent spread as a pandemic, triggered widespread disruptions and turmoil across the world [24]. The economic impacts are particularly severe because most business leaders apparently failed to anticipate and prepare for a global pandemic. As a result, logistics, operations and supply chains have been stretched to their breaking points [25]. Authorities in many localities responded to the initial surges of the disease by implementing lockdowns that imposed mass quarantines and temporarily shuttered non-essential businesses [26,27]. Subsequently, in response to latter secondary surges of the pandemic, additional periods of lockdowns in many places with varying levels of restrictions were imposed throughout the rest of 2020 [28,29]. Public health attempts to control the pandemic also include the use of regional lockdowns, home quarantines, travel restrictions, and business closures [27,30,31]. Policies enacted by the U.S. Department of Homeland Security were used to determine if businesses are part of essential infrastructure that should remain open during the crisis, or else be deemed as non-essential infrastructure which have to be closed [32]. In California, the automated test companies were regarded as non-essential businesses, and were subject to constraints that hamper, or in some cases, halted, their ability to conduct field-tests of automated prototypes during these periods [33].

In mid-March 2020, California was the first US state to announce and to implement a statewide shelter-in-place order or SIPO that shuts non-essential businesses, and curtails non-essential travel [34]. These restrictions act to directly reduce the labor availability of companies across supply chains. Companies that are developing automated vehicles are regarded as non-essential businesses in California and for them, the effects of the shutdown were immediate. They ceased their testing operations, sent most employees home, and at most, kept a core group of employees onsite only for office administration tasks [35–37]. Aside from having to comply with government mandates, these companies, like all others, also need to address the multiple concerns of their employees, some of whom do not want to risk themselves or their families with exposure to the virus in workplaces, while others are anxious to keep working regardless of public health restrictions to continue to collect their paychecks. In some companies, employees were laid off to collect unemployment pay during the shutdown period, while in others, employees were kept on the payroll but encouraged to perform volunteer Covid-19 related relief efforts [23,35,38–40]. In all cases, the ability of these companies to continue prototype field testing was severely constrained during these periods.

This paper seeks to identify and analyze the extent in which the testing stage of the technological development process of automated vehicles is affected by the Covid-19 pandemic, and also if the effect will persist in the immediate aftermath of the crisis. The remainder of the paper is organized as follows; the next sections cover the literature review, data, methods, and tests used to analyze changes to test mileage trends, test operation resumptions, and the test environment due to the pandemic together with their results and analyses; and is followed by the discussion section which reviews the results; and finally, the limitations and conclusion sections.

## Literature review

Even from early times, people have to sought to automate the regular operations of their vehicles. As far back as the era of steam-engines in the nineteenth century, inventors had designed mechanical controls that automatically maintain a steady engine throttle so that trains may run and operate without constant human intervention [41]. In the early twentieth century, Sperry demonstrated the first aerial auto-pilot that used a combination of gyroscopes to automatically keep airplanes on a steady course in flight [42,43]. In due course, mechanical auto-pilots or cruise controls become available to automated some of the simpler driving operations of other vehicles such as ships and cars [44–46]. Studies to further automate road transportation began in the 1950’s with experiments made using automobile mock-ups with sensors that ran on test tracks equipped with embedded control guiding wires [47,48]. It is apparent that transportation experts have always believe that the use of automated vehicles can increase logistics efficacy by reducing congestion, increasing fuel savings, reducing pollution, and improving road safety for all users [49]. However it was not until towards the end of twentieth century, when sufficient computing power become available at reduced sizes and costs, that more sophisticated automated driving systems with electronic sensors and negative feedback computer controls emerged that could be installed into vehicles [50,51]. In the 1990s, the first laser-based proximity detection and warning systems in automobiles became available for general use [52–54]. Later developments brought multiple types of sensor-based cruising systems, variously referred to as "autonomous", "automated", "intelligent" or "adaptive" systems, that could automatically keep to lanes, follow other vehicles, or brake as needed without further driver input [52,55–58].

Recent studies in automated driving systems have addressed more complicated road situations such as disturbances to the traffic flow caused by weaving, deviating, cutting-in and cutting-out, lane changing, and ramp exiting driving behaviors, and includes the development of optimal lane change strategies for automated vehicles by predicting the impacts of lane changes [59–61]. Recent studies include research into more efficient transportation techniques such as cooperative driving in platoons [62–64]. The use of platoons can greatly reduce road congestion and increase safety by tight spacing between vehicles within platoons, while also improving efficiency via aerodynamic drag reduction for each platoon [65]. Cooperative platoons are made possible by the ability of automated vehicles to stay interconnected via vehicle-to-vehicle (V2V) communications and to react automatically to changes to their driving environment. Controlled cooperative and learning behavior among vehicles can serve to reduce the propagation of random fluctuations or oscillations in traffic to increase efficiency [62,66,67]. These studies has in turn lead to further research into the reliability of information propagation, and the consequences of any communication failure, delay or coverage, among cooperating vehicles [68–73]. Research has also been made into automated vehicles safety performance through the use of surrogate safety measures [74]. Of particular relevance to this paper, the risks of rear-end collisions to automated vehicles has also been highlighted in the literature with proposed remedies [75]. This paper seeks to further contribute to this literature that investigates the ongoing technological development process of automated vehicles.

## Sample data

The California DMV website contains publicly available records of the total autonomous test mileage per month reported by all past and present automated vehicles test companies registered in California. The DMV website cut-off date for data collection in each year is November 30^th^. As such, this study measures annual reporting data as starting on December 1st of the preceding year to November 30^th^ of the next year. For example, the 2015 reporting year covers the period from December 2014 to November 2015, while the 2020 reporting year covers the period from December 2019 to November 2020. For the purpose of this study which compares test operations year by year, it is necessary to exclude 29 companies registered in California that either did not, or only partially conduct test operations in 2019 and/or 2020. That leaves 22 companies in the study sample. We further obtain data from these companies to test their test mileage trend, to examine their test operations resumption patterns, and the detect any change to their test environment due to the pandemic [76,77].

## Test methods and results

### Test mileages trend

**Fig 1:**
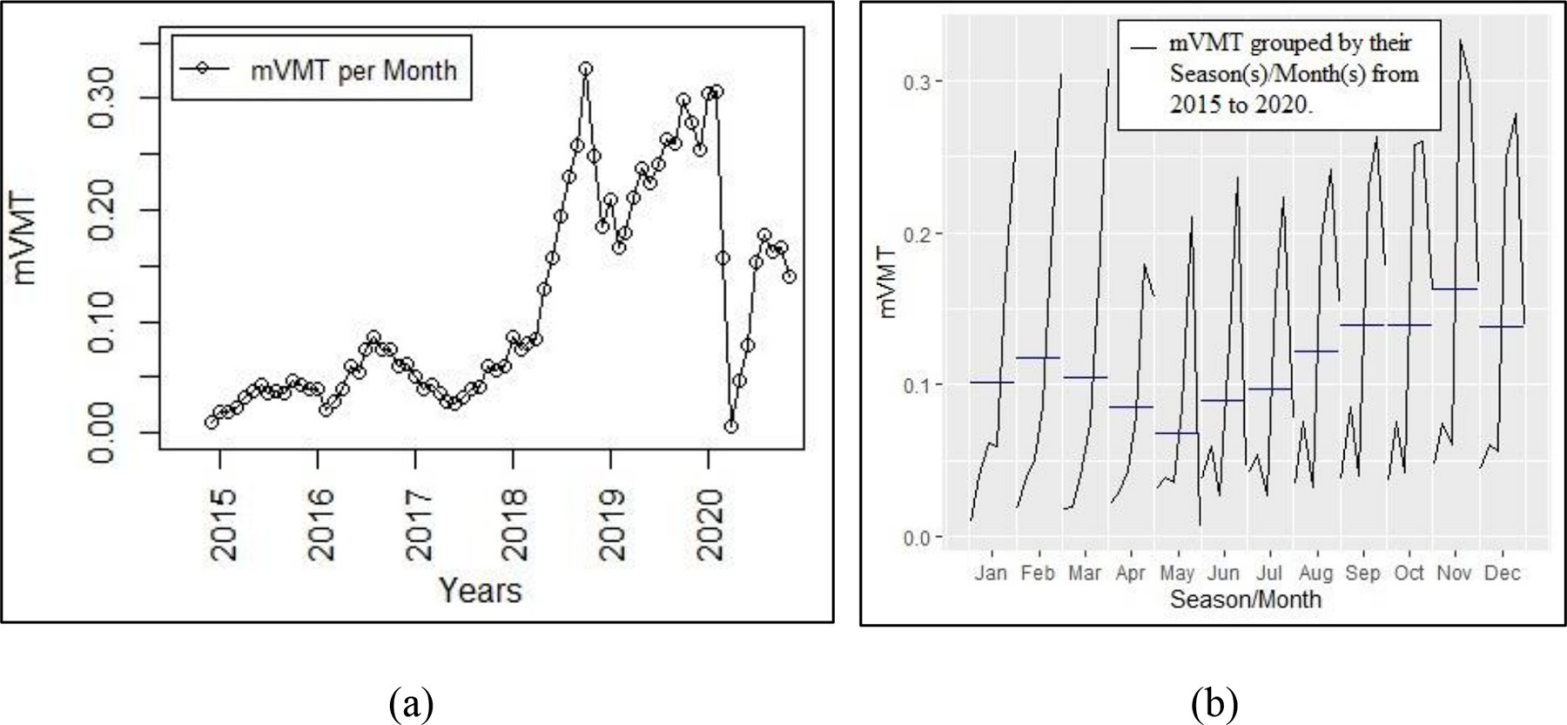
(a) Overall Time-Plot (mVMT), and (b) Plots by Season(s)/Month(s) (mVMT).

The total autonomous test mileage of the selected sample of these 22 companies amount to 8.23 mVMT, or Vehicle Miles Traveled in millions of miles, which is 96.5% of the total reported test mileage of all test companies, past or present, reported in the DMV website [78]. The time-plot in Fig 1(A) shows the total autonomous test mileage per month reported by these 22 automated vehicles companies from December 2014 to November 2020.

Fig 1(A) diagram shows that, when compared with earlier years, there was a sharp drop of overall test mileage in early 2020. Fig 1(B) shows this effect more clearly by grouping the test mileage for the same month in each year from 2015 to 2020. This creates a sub-seasonal plot that shows how the test mileage drop-off starts in March 2020, and continues to November 2020. Table 1 breaks down test mileage data by companies and years, with the top 5 companies with highest total test mileage shown. The data in the table shows that each test companies have different mileage responses to the pandemic shutdowns. For instance, compared to 2019, Waymo, GM Cruise and Nuro have reduced mileages in 2020, while Pony.ai and Zoox have increased mileages.

**Table 1:** Test companies mileages by reporting year (mVMT).

	Waymo	GM Cruise	Pony.ai	Zoox	Nuro	Others (17)	Totals
**2015**	0.380	0.000	0.000	0.000	0.000	0.002	**0.383**
**2016**	0.636	0.010	0.000	0.000	0.000	0.005	**0.651**
**2017**	0.353	0.132	0.001	0.002	0.008	0.019	**0.515**
**2018**	1.243	0.448	0.016	0.028	0.025	0.168	**1.928**
**2019**	1.454	0.831	0.175	0.067	0.069	0.154	**2.750**
**2020**	0.629	0.770	0.225	0.103	0.055	0.172	**1.955**
**Totals**	**4.695**	**2.190**	**0.418**	**0.200**	**0.157**	**0.521**	**8.181**

A test is performed to determine if the mileage drop-offs in 2020 shown in Fig 1 are statistically significant. We first need to explore if the mileage data (mVMT) reported by the companies has a seasonal component, as seasons need to be accounted for when testing for trend to avoid confounding the two components. Seasonality is a reasonable assumption, given that normal road travel is subject to climate or holiday effects. For instance, road test operations can be more constrained by seasonal bad weather conditions, or be more unrestricted during seasonal dry or sunny weather. Seasonal effects such as summer travel congestion or increased numbers of buses during the school year can also affect the ability to conduct test operations. Test companies will also normally not operate on regular holiday dates that repeat on an annual basis. We use the *findfrequency()* function from the r package “*fpp2*” to detect seasonal periods in the time-series data for the top 5 companies. We use data reported before the reporting year 2020, i.e., data from Dec 2014 until Nov 2019, to determine the seasonal components that may exist in the pre-Covid era. Earlier observations are truncated from the data sets in order to allow the time-series to reach stability. The results, not shown here, suggest the existence of annual seasonal patterns close to 12, or multiples of 12, seasons per year. Based on this breakdown, it will seem reasonable to assume that these data sets exhibit 12 seasons in each year, and that these are based on the 12 months of each year.

Next, we use the *kendallSeasonalTrendTest()* function from the *r* package “*EnvStats*" to detect if monotonic trends are present in the overall dataset that comprise mileage data from the test companies in our sample, operating under the above assumption that a monthly seasonal component exists. We first perform the test for the “*pre-Covid era*” for mileage observations from Dec 2014 to Nov 2019 (60 monthly observations) to examine if there are any trend during that period. In this test, there is a general assumption that all seasons of a dataset must have the same trend direction for this test to be able to provide valid results. To test that this assumption holds for a particular dataset, the *kendallSeasonalTrendTest()* function first performs a chi-square test to determine if all seasons shows the same trend direction as per the test for trends across seasons as first described by Van Belle and Hughes [79]. The null hypothesis for the chi-square test is that all seasons shows the same trend direction. The *kendallSeasonalTrendTest()* function next checks if there is an overall monotonic trend by determining the numbers of concordant versus disconcordant pairs independently for each season and using these to calculate a *z* statistic. The function will then return estimates for the values of Kendall’s tau, the slope, and the intercept for each season, as well as a single estimate for each of these three quantities combined over all seasons. The null hypothesis for this *z* test is that there are no trends in the sequence of observations, and the alternative hypothesis is that there is a monotonic trend in the sequence.

If we can establish that a trend exists for test mileages prior to the Covid-19 pandemic, we are next interested to determine if the onset and occurrence of the pandemic affects this known pattern. We then perform the Kendall seasonal trend test for the “*immediate pre-Covid plus Covid era*” for mileage observations from Dec 2018 to Nov 2020 (24 monthly observations) to estimate if a trend also exists over that period.

### Test mileages trend results

Our “*pre-Covid era*” chi-square test results shown in Table 2, (χ^2^ = 1.660; p-value = 0.999), suggest that we should fail to reject the above null hypothesis, and conclude that the seasons do show the same direction in their trends. The “*pre-Covid era*” z-trend test results shown in Table 2, (overall Kendall’s tau = 0.783; p-value = 4.83e-11), suggests we should reject the above null hypothesis, and to conclude that a significant monotonic and positive trend in mileage is present for test vehicle operations during this era.

**Table 2:** Kendall seasonal trend test results.

Test Period	χ^2^	z (Trend)	Kendall’s tau	Slope	Intercept
Pre-Covid Era (60 months)	χ^2^ = 1.660; (p-value = 0.999)	z = 6.576; (df = 11; p-value = 4.83e-11)	0.783	0.045	-90.98
Immediate Pre-Covid plus Covid Era (24 months)	χ^2^ = 9.00; (p-value = 0.622)	z = -1.443; (df = 11; p-value = 0.149)	-0.500	-0.092	186.94
Extended Pre-Covid plus Covid Era (36 months)	χ^2^ = 12.00; (p-value = 0.364)	z = 0; (df = 11; p-value = 1.0)	0	-0.0091	65.99

The “*immediate pre-Covid plus Covid era*” z-trend test results shown in Table 2, (overall Kendall’s tau = -1.443; p-value = 0.149), suggests we should not reject the null hypothesis, and to conclude that no monotonic trend exists in that era. As a check, a follow-up Kendall seasonal trend test for the “*extended pre-Covid plus Covid era*” for mileage observations from Dec 2017 to Nov 2020 (36 monthly observations) was performed that also provided the same conclusion as shown in Table 2. These tests suggest that the onset of the pandemic in early 2020 did significantly affected the test mileages for the industry by ending the formerly monotonic and positive trends found in their test mileages.

### Test operations resumption

The 22 companies in the sample dataset are independently operated entities, and as such they typically should not act as a single monolithic bloc in response to any event, including to the pandemic. We take a closer look at each company to compare and contrast their reported test mileages for the “Immediate Pre-Covid Era” against the “Covid Era”. We observe their respective test mileage time plots to compare and categorize their responses in resuming test operations while the pandemic is still ongoing. The graphs and our observations are reported below.

### Test operations resumption results

**Fig 2:**
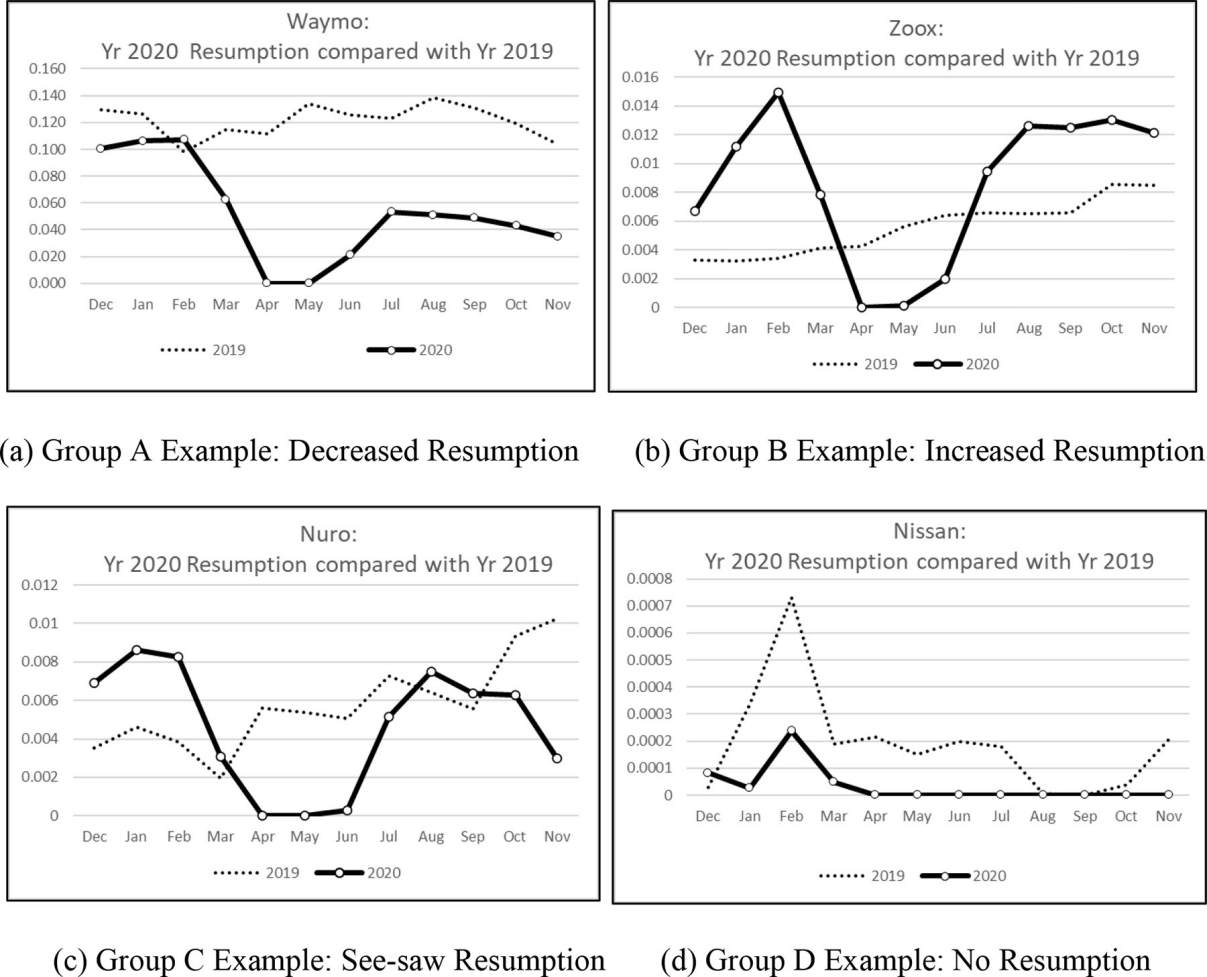
Test Mileage Resumptions of four example companies each respectively drawn from Groups A, B, C and D (mVMT). (a) Group A Example: Decreased Resumption (b) Group B Example: Increased Resumption (c) Group C Example: See-saw Resumption (d) Group D Example: No Resumption.

The graphs of test mileages reveal that the companies in our sample fall into at least four different groups based on their test resumption pandemic responses. We identify these as Groups A, B, C and D. Fig 2 shows one example taken from each of these four groups that show the plots of their respective test mileages (mVMT) in 2020 compared with 2019. The example diagrams in Fig 2 illustrates how the test companies from all groups initially complied in the same way to the shutdowns of March or April 2020 by a general cessation of their test operations. As lockdown requirements change over the months, the companies subsequently enacted different test operations responses as they respectively re-strategize test goals, interpret lockdown restrictions, or adjust or negotiate new labor needs.

Group A companies are those that temporarily halt all testing at the pandemic onset. Afterwards, these companies resume test operations but at a consistently reduced level compared to the Immediate pre-Covid era. This group consists of 9 companies, i.e., Waymo, GM Cruise, Pony.ai, Aurora, Didi Research America, Lyft, AI Motive, and AutoX. It is possible that these are companies that have already accumulated sufficient test records, or believe that their ADS technology is sufficiently mature, and so are satisfied with a reduced level of test operations given the ongoing risks of the pandemic. Alternatively, it is also possible that these companies continue to have labor or other operational constraints due to the pandemic that limited their ability to fully resume operations.

Group B companies temporarily halt all testing at the pandemic onset but then follow that with an increased resumption of test operations. Their test mileage totals for each month are generally higher as compared to the Immediate pre-Covid era mileages. This group consists of 3 companies, i.e., Zoox, WeRide, and Valeo. These could be companies that needed to catch up with lost time, and seek to boost their test operations after the initial shutdown period. It could also mean that these companies are able to overcome pandemic hurdles to achieve higher test operation mileages.

Group C companies also temporarily halt all testing at the pandemic onset, but afterwards follow a see-saw resumption of test operations. Their test mileage totals for different months during the “Covid Era” can fluctuate to be either above or below those of the corresponding months from 2019. This group consists of 8 companies, i.e., Nuro, Mercedes-Benz, Apple, Nvidia, Gatik, Toyota Research Institute, Qualcomm Technologies, and SF Motors. These companies could be showing variable levels of test mileages because they may attempt to resume normal test operations, but they may not always succeed in doing so in every month due to on-going pandemic-related reasons such as labor or resource constraints.

Group D companies are those that come to a complete halt to testing at the pandemic onset, and do not resume test operations for the remainder of the reporting year. This group consists of 3 companies, i.e., Nissan, Telenav, and Udelv. These companies will likely have different reasons for not resuming operations, and these may range from a deliberate decision to stop testing to sit out the pandemic, to not being able to resume testing at all due to continuing pandemic-linked constraints.

### Test environment change

Another important aspect to consider is if there is any change to the test environment for these companies given the novel conditions of the pandemic. For instance, traffic patterns were reported to have changed in many cities due to the pandemic, resulting in reduced congestion, higher overall speeds, and shifts to rush hour timings due to permanent changes to work schedules [80,81]. We wish to determine if any such change to the road environment will affect the test operation results of autonomous vehicles.

A characteristic of test operations conducted on public roads is that an autonomous vehicle may occasionally be involved in an accident. Accidents are not planned, but can be regarded as a random event that occasionally emerge from the intersection of the functions of the ADS technology, and the road environment [82]. We assume that the ADS technologies among the test companies generally improve over time through software debugging, or hardware upgrades, and as such accidents which did occur as a function of ADS technologies will normally decline over time. We also assume that the road environment is normally consistent all the time, and as such, the numbers or types of accidents which occur as a function of the road environment should similarly be consistent over time. However, this assumption should no longer hold if the road environment significantly changes for any reason.

Our observations of reported accidents involving autonomous vehicles show that a considerable number of rear-ending accidents occur to them. Rear-ending accidents are of special interest for a number of reasons. First, the collision records clearly identify this type of accident whenever it occurs. The damage listing or description in the accident report is typically unambiguous, so it is unlikely for the records to confuse this particular type of collision with other types of accidents. This makes rear-ending accidents clearly identifiable as a unique collision type in the dataset. Second, fault is relatively easy to assign because for situations when vehicles are proceeding normally on the roadway, a vehicle that hit the rear of another vehicle is generally the one at fault [83]. As such, we can generally assume that an autonomous vehicle is not at fault if it is accidently hit from the rear by other vehicle during normal road operations. This helps to rule out the general case of accidents caused by automation technology errors [84]. Next, if we assume that the driving operations quality of autonomous vehicles are consistent or can only improve over time, and that if all other factors remain the same, then any change to the ratio of rear-ending accidents caused by other vehicles over time could only be attributed to changes to the driving norms of human drivers in the surrounding road environment [85]. In this section, we analyze and compare rear-ending accident ratios that occur during test operations as a proxy to determine the nature of changes to the road environment due to the pandemic conditions.

The time periods used in the preceding sections were based on mileage reporting years which end on the November 30^th^ of each year. That factor had previously limited our latest data point to November 30^th^, 2020. In this section, as we are viewing collision reports which are usually posted very soon after each occurrence, so we are able to obtain these reports up to May 31^st^, 2021. Various forms of the Covid-19 restrictions have been ongoing since March 19^th^, 2020 to the present in California [86]. We identify the “Covid-Restricted” period to be from March 19^th^, 2020, to May 31^st^, 2021, or a period of 439 days. Based on this, we counted backwards for 439 days from March 19^th^, 2020, to January 5^th^, 2019, and define this as the “Non-Restricted” period, which we will use to compare against the “Covid-Restricted” period.

The California DMV site listed 311 autonomous vehicle collision reports from test operations with drivers recorded from September 2014 to end-May 2021 [22]. From these publicly available reports, we exclude 114 incidents as these involved accidents that occur when the autonomous vehicles were operating in “conventional” mode, i.e., when they were being directly controlled and operated like ordinary vehicles by human drivers, and so fall outside the category of vehicles being autonomously operated. We also excluded one earlier accident that involved a test company that did not subsequently operate during the reporting year 2020; another five accidents in which the autonomous test vehicles are at fault as these belong to the category of accidents related more to ADS technologies and not strictly to the road environment; and five more accidents in which the faults were not caused by other road vehicles, but were variously caused by random factors such as pedestrians, skateboards, and a golf ball. This leaves 186 accidents in total which involve an autonomously operated test vehicle, and where another road vehicle which is at fault for the accident. Our final sample is obtained by considering only accidents that occur during the “Non-Restricted” and the “Covid-Restricted” periods. These added up to 105 collisions, and their breakdown into non-rear-ending and rear-ending accidents are shown in Table 3 [87,88].

**Table 3:** Accident types during Non-Restricted vs Covid-Restricted periods.

	Non-Restricted	Covid-Restricted	Totals
Non-rear-ending accidents	45	8	**53**
Rear-ending accidents	32	20	**52**
**Totals**	**77**	**28**	**105**

A face observation of the data suggests that the ratio of rear-ending accidents increases during the “Covid-Restricted” period as compared with the earlier period. We apply Boschloo’s Test for Count Data using the “*boschloo*” function from the r package “*exact2x2*” to determine if a significant difference exists between the ratios of non-rear-ending accidents versus rear-ending accidents compared between these two periods. We apply the one-sided test where the *null hypothesis* is that the ratio during the “Covid-Restricted” period is less than the ratio during the “Non-Restricted” period.

### Test environment change results

The result of the test on the ratios of non-rear-ending versus rear-ending accidents between the two periods (data: x_1_/n_1_ = (32/77) and x_2_/n_2_ = (20/28); proportion 1 = 0.416, proportion 2 = 0.714; *p*-value = 0.00348 or 0.348%) suggests that we should reject the null, and concludes that the ratio of rear-ending accidents did significantly increase during the “Covid-Restricted” period for the test companies in our sample in Table 3.

We further breakdown the accident data for two test companies with the highest test mileages in our sample, i.e., Waymo and GM Cruise, to examine this effect with respect to individual companies. Their collision records are shown in Tables 4 and 5, respectively.

**Table 4:** Waymo accident types during “Non-Restricted” vs “Covid-Restricted” periods.

	Non-Restricted	Covid-Restricted	Totals
Non-rear-ending accidents	8	1	**9**
Rear-ending accidents	13	7	**20**
**Totals**	**21**	**8**	**29**

**Table 5:** GM cruise accident types during “Non-Restricted” vs “Covid-Restricted” periods.

	Non-Restricted	Covid-Restricted	Totals
Non-rear-ending accidents	34	5	**39**
Rear-ending accidents	16	8	**24**
**Totals**	**50**	**13**	**63**

We apply Boschloo’s Test for Count Data to the Waymo data shown in Table 4, and the test result (data: x1/n1 = (13/21) and x2/n2 = (7/8); proportion 1 = 0.619, proportion 2 = 0.875; p-value = 0.119 or 11.9%) suggests that we should fail to reject the null, and conclude that the accident ratio of rear-ending accidents for Waymo did not significantly differ from one period to the other. A face observation of the data in Table 4 suggests that there is some difference to the ratio, but not markedly so. Alternatively, the sample size for the Waymo test in each category may be insufficient at this time to confirm significance. We next apply the test to the GM Cruise data shown in Table 5, and the test result (data: x1/n1 = (16/50) and x2/n2 = (8/13); proportion 1 = 0.32, proportion 2 = 0.615; p-value = 0.0298 or 2.98%) suggests that we should reject the null. For this test company the accident ratio is significantly different across the periods, and that the rear-ending accident ratio substantially increased during the “Covid-Restricted” period.

## Discussion

This study determines that the pandemic significantly affect test operations that are important to the development of ADS technology. Measured on a seasonal basis, overall test mileages significantly dropped. This could mean that the companies will have reduced opportunities to test real-world scenarios against new software or hardware fixes. The reduction in test opportunities may affect new test companies just entering into the ADS industry more than more established players. Such reductions likely occur because the pandemic creates labor issues when experienced employees, including test drivers, were either laid off, or elect to enter alternative employment. The absence of such staff will negatively affect current and future test operations. Differences to the companies’ test resumption patterns also suggest that the pandemic stress has hasten the competitive shakedown in the ADS industry. The development constraints due to the pandemic may cause some test companies to permanently exit the industry, and curtail their future participations in the development of ADS technologies.

The pandemic also creates significant change to the road test environment, notably to traffic patterns and more erratic or distracted driver behaviors. In the long run, this change may act to benefit the development of ADS technologies. The sudden increase to the ratio of rear-ending accidents shows that some ADS technologies may still need further refinements to handle road situations with increased variances to traffic density or speed as may have occurred during the pandemic. The road environment in California may eventually adjust back to more normal conditions after the end of the pandemic, but these erratic road conditions might still reappear in the event of future crises. ADS technologies that are already geared to handle such road situations will have an advantage going forward. The mixed results from the rear-ending accident study, where different companies encounter different rear-ending accident ratios in a changed road environment, indicate that some companies may already be able to handle shifting road environments better than others. Driving norms can apparently shift quickly in response to crisis situations, and ADS technologies may need to be designed to more quickly detect and switch to match new social driving norms [89,90].

## Limitations

The data for this study is limited to records collected from the California test program. The State of California is regarded as an attractive location for new technology development due to the size of its information technology sector, automation-friendly regulations, and future market potential. However, the necessity to provide public test records in California may have deterred some test companies from conducting their tests there, and to seek test locations in other states or countries. It is possible that test operations in other areas could have different pandemic responses due to differences to local lockdown laws. An inspection of the postcodes of reported collisions suggests that most test operations take place within a few counties centered around San Francisco. The road environment there is fairly diverse with a mix of urban and suburban streets and highways. However, it cannot be representative of every other road environment to be found. Social driving norms are also well-known to be very localized, so changes to norms and changes to accident ratios due to the pandemic may not be the same in other parts of the world. In the same way, the climate of California, which can affect the conduct and results of test driving operations, also cannot be generalized to all climates.

## Conclusion

This study focuses on the pandemic effects to the test development of automation technologies for road transportation. Road transportation covers personal travel, as well as short and long haul freight connections, and is the most flexible mode in supply chain logistics. It is also very important as an intermodal connector for freight, and indispensable to last mile deliveries. The test companies in our study are developing ADS technologies which can equally be deployed for personal use, or for hire, or for freight shipments, in future logistics.

The Covid-19 pandemic has made clear that that the most vulnerable part of supply chain logistics is the human operator. Even at the best of times, human beings exhibit high variability in performance, and provide a limiting constraint to scheduling in logistics. During health emergency lockdowns, traditional logistics infrastructure may still be undamaged and available, but none of them will function in the absence of a trained human operator. While the development and capital costs of automation are high, their operational and overhead costs are significantly lower, and their utilization rates can be much higher. Our current supply chain management logistics capabilities still require a substantial increase in the automation component of road transportation if the disruption effects of future pandemics are to be mitigated. It is unfortunate that the pandemic itself has acted to hobble such automation efforts by reducing the ability of the test companies to conduct tests, or to retain test employees. This effect continues during the immediate aftermath of the initial lockdowns because many employees choose not to return to previous logistics jobs over concerns of continuing exposure to new variants of the disease, and many companies also did not hire or train new employees during their pandemic hiatus.

Public policies in operation during the pandemic contributed to problems faced by these companies. It is often difficult for governments to enact public interest policies that appropriately balance the needs of public health versus the needs of businesses to operate [91,92]. The lockdown policies used in California are based on those published by the Cybersecurity and Infrastructure Security Agency (CISA), a part of the U.S. Department of Homeland Security, which identify critical infrastructure workers deemed essential that must continue operations during emergencies, versus non-essential ones that must cease operations [33]. Understandably, CISA policies place priority on maintenance, repair, and operational activities that will keep vital and everyday infrastructure functioning rather than on research and development activities that will only have payoffs in the future. For instance, Transportation and Logistics is identified as essential infrastructure but only for support, repair, commercial, continuity, or emergency services. The CISA policies identify research and development efforts as essential in only two areas; namely, Healthcare, and also in Information Technology [32]. Automated vehicles research and development is an emerging industry that overlaps both Transportation and Logistics as well as Information Technology areas, and perhaps as a consequence, was overlooked as constituting a part of vital infrastructure. Given the importance of advancing automation in logistics to mitigate supply chain pandemic problems, it is apparent that it is necessary to continue research and development efforts in this area even during health emergencies. Future public policies should be directed to aid these test companies and to recognize their workforce as essential infrastructure workers, to enable them to maintain development efforts to develop, build and safeguard the automation capabilities of future road transportation.
